# Rapid Extraction Method of *Mycobacterium ulcerans* DNA from Clinical Samples of Suspected Buruli Ulcer Patients

**DOI:** 10.3390/diagnostics9040204

**Published:** 2019-11-26

**Authors:** Michael Frimpong, Hubert Senanu Ahor, Samuel Asamoah Sakyi, Bernadette Agbavor, Emmanuel Akowuah, Richard Odame Phillips

**Affiliations:** 1 Kumasi Centre for Collaborative Research in Tropical Medicine, Kwame Nkrumah University of Science and Technology, Kumasi AK 312, Ghana; hubertsenanu@gmail.com (H.S.A.); agbavor@kccr.de (B.A.); akowuah@kccr.de (E.A.); phillips@kccr.de (R.O.P.); 2 Department of Molecular Medicine, School of Medical Sciences, Kwame Nkrumah University of Science and Technology, Kumasi AK 312, Ghana; samasamoahsakyi@yahoo.co.uk

**Keywords:** *Mycobacterium ulcerans*, PCR, recombinase polymerase amplification, point-of-care

## Abstract

Isothermal amplification techniques such as recombinase polymerase amplification (RPA) and loop-mediated isothermal amplification (LAMP) for diagnosing Buruli ulcer, a necrotic skin disease caused by *Mycobacterium ulcerans*, have renewed hope for the molecular diagnosis of clinically suspected Buruli ulcer cases in endemic districts. If these techniques are applied at district-level hospitals or clinics, they will help facilitate early case detection with prompt treatment, thereby reducing disability and associated costs of disease management. The accuracy as well as the application of these molecular techniques at point of need is dependent on simple and fast DNA extraction. We have modified and tested a rapid extraction protocol for use with an already developed recombinase polymerase amplification assay. The entire procedure from “sample in, extraction and DNA amplification” was conducted in a mobile suitcase laboratory within 40 min. The DNA extraction procedure was performed within 15 min, with only two manipulation/pipetting steps needed. The diagnostic sensitivity and specificity of this extraction protocol together with *M. ulcerans* RPA in comparison with standard DNA extraction with real-time PCR was 87% (*n* = 26) and 100% (*n* = 13), respectively. We have established a simple, fast and efficient protocol for the extraction and detection of *M. ulcerans* DNA in clinical samples that is adaptable to field conditions.

## 1. Introduction

*Mycobacterium ulcerans* (*M. ulcerans*) is the causative agent of Buruli ulcer (BU), a deforming skin disease mostly reported in rural communities in most endemic countries. Early diagnosis is critical in BU case management, as antibiotic treatment is very effective with a combination of rifampicin and clarithromycin/streptomycin [1,2]. The Gold standard diagnostic technique for BU is polymerase chain reaction (PCR) targeting the IS*2404* insertion sequence of *M. ulcerans*. The deployment of this technique in endemic communities is hindered due to the sophistication required in setting up and logistical constraints of endemic communities. As a result, PCR is performed in reference laboratories far away from endemic communities [3,4], resulting in delays in initiating treatment.

Recombinase polymerase amplification (RPA) [3] and loop-mediated isothermal amplification (LAMP) [5,6,7] have been developed as diagnostic tools for BU. These techniques were proposed as field-friendly diagnostic techniques during a diagnostic conference organized by the Foundation for Innovative New Diagnostics (FIND) and World Health Organization/Neglected Tropical Diseases (WHO/NTD) [8] due to their high sensitivity (>85%) and specificity (100%). However, the use of these amplification methods as well as other molecular techniques at point of need requires simple and rapid DNA extraction techniques which can effectively extract *M. ulcerans* DNA from clinical specimens without affecting the diagnostic performance of these amplification techniques [3,5,7].

Different commercial kits as well as in-house extraction techniques have been employed in the extraction of *M. ulcerans* DNA from environmental or clinical samples [9,10,11,12]. These methods employed the use of chemical lysis, enzymatic lysis and physical disruptions with or without DNA purification [13,14]. Certain commercial kits, particularly Puregene Genomic DNA purification kits, together with enzymatic digestion with proteinase K have been employed routinely in the extraction of *M. ulcerans* DNA from tissue biopsy, fine-needle aspirates (FNAs) and swab samples from BU patients successfully [15,16,17]. Notwithstanding, these extraction techniques are time consuming, complex and can only be performed in well-equipped laboratories and not at point of care.

In order to provide a diagnostic platform for suspected BU cases at point of need, we describe a rapid extraction protocol (Mu DNA GenoLyse) based on the use of GenoLyse^®^ reagents (Hain Lifescience GmbH, Nehren, Germany). The destruction of *M. ulcerans* cell wall depends on the chemical lysis of cells when heated. The inclusion of a centrifugation step allows the pelleting of cell debris as well as other contaminants. This extraction technique will enhance the performance of our earlier developed *M. ulcerans* RPA and LAMP assays in the field.

## 2. Materials and Methods

### 2.1. Clinical Samples

Fifty-eight samples comprising 25 fine-needle aspirates (FNAs) and 33 swabs were collected from BU lesions in clinically suspected patients during a routine visit to BU treatment centers at Agogo Presbyterian Hospital, Dunkwa and Tepa Government hospitals in Ghana. The diagnosis of BU patients was confirmed by IS*2404* real-time PCR [17,18].

### 2.2. M. ulcerans Genolyse DNA Extraction Protocol

**Figure 1:**
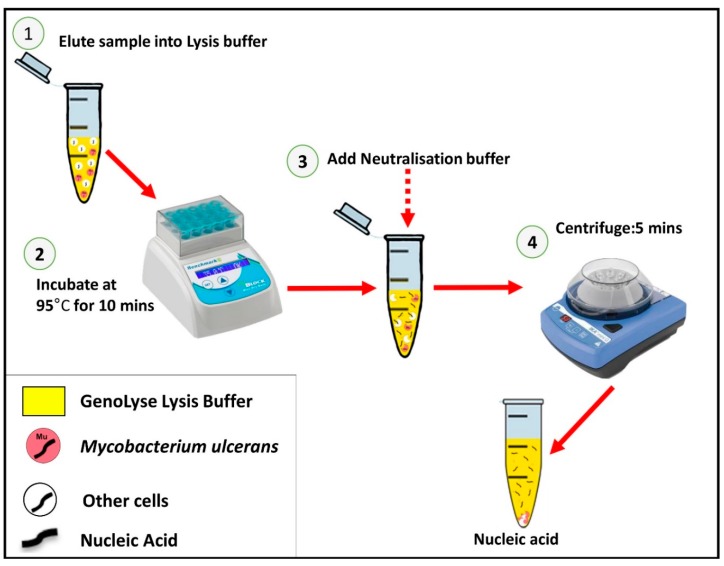
Workflow of the sample and rapid *Mycobacterium ulcerans* (Mu) DNA GenoLyse extraction protocol. The whole extraction procedure is performed in approximately 15 min.

To facilitate a fast but effective DNA extraction, fifteen (15) clinical samples were extracted with both a Puregene (Qiagen, Hilden, Germany) and GenoLyse^®^ DNA extraction Kit (Hain Lifescience GmbH, Nehren, Germany). Two samples per patient were pooled together in 700 µL of phosphate buffer saline (1× PBS). Samples were vortexed and centrifuged at high speed for 5 min and supernatant discarded. Pellets were resuspended in 30 µL 1× PBS and divided into 2 tubes—15 µL for each extraction method. One part was extracted with the standard Puregene DNA purification method described previously [16,17] and the remaining 1 part was extracted using a GenoLyze Kit (Hain Lifescience GmbH, Nehren, Germany) according to the manufacturer’s protocol, with some modification to suit our purpose. The simple fast Mu DNA GenoLyse^®^ lysis protocol (Genolyse kit, Hain, Germany) was deployed as follows: samples (FNA or swab) were eluted in 100 or 200 µL lysis buffer respectively and incubated at 95 °C for 10 min. An equal volume of neutralization buffer (100 or 200 µL) was added to FNA or swab samples, respectively, briefly vortexed and centrifuged at 3000 rpm for 5 min. A schematic diagram of the process is a shown (Figure 1).

The quality and quantity of extracted DNA were determined by DeNovix DS-11 Spectrophotometer. Five microliters of DNA extract was amplified by Mu RPA assay [3] in a 50 µL reaction volume and 2 µL with real-time PCR in a 20 µL final reaction volume as described previously [17,18].

### 2.3. RPA Assay

The RPA assay was conducted as published previously [3]. Briefly, 2.1 μL of 10 μM of both forward (5′-*ATG CAT CGC ATC CAC AGT GAC CAG CCA CCG-3*′) and reverse primer (5′-*ATT GGT GCC GAT CGC GTT GGA CGG CAA GAT G-3*′), 0.6 μL of 10 μM Probe (5′-*GTA GGC GAA CAC CGA CAC GAG ATG CGT GGC* BHQ1-dt, Tetrahydrofuran and Fam-dT (F) *CGC TTT GGC GCG TA – PH-3*′), 29.5 μL of rehydration buffer, 8.2 μL DNAse-free water, 5 μL of the DNA template and 2.5 μL 280 mM Magnesium acetate (MgAc) were added to a lyophilized RPA reaction pellet (TwistDx Exo kit, Cambridge, MA, USA). The RPA reaction was run for 15 min at 42 °C and fluorescence signals were read with Axxin T8-ISO fluorometer (Axxin Pty Ltd., Victoria, Australia).

### 2.4. Clinical Sensitivity and Specificity of Mu DNA GenoLyse RPA Assay

The performance of the Mu DNA GenoLyse protocol together with the Mu RPA assay (Mu DNA GenoLyse RPA) was evaluated with 43 clinical samples from suspected BU cases. Four samples (two for RPA and two for PCR assay) were taken for each patient following the WHO recommended guideline for sample collection. DNA extraction with Mu DNA GenoLyse protocol and amplification with Mu RPA assay were performed in a mobile laboratory suitcase [19,20]. The results of the Mu DNA GenoLyse RPA assay were compared with the results of the Puregene DNA extraction (Qiagen, Hilden, Germany) with IS*2404* real-time PCR.

### 2.5. Statistics

Patient data and experiment results were entered into Microsoft Excel 2016 and analyzed using GraphPad Prism v.6 (GraphPad software, San Diego, CA, USA). General descriptive information of patients such as frequency, percentages, median and interquartile ranges were determined with descriptive statistics. A Mann–Whitney test was used to compare the quality and quantity of DNA extracted with Gentra Puregene extraction and GenoLyse^®^ DNA extraction kit. A contingency table was employed to calculate the sensitivity, specificity and the predictive values of Mu DNA GenoLyse RPA using Puregene IS*2404* real-time PCR as the gold standard.

### 2.6. Ethics Statement

Ethical approval for this study was given by the Committee on Human Research, Publication and Ethics (CHRPE/AP/122/17, on 28 February 2017) at the School of Medical Sciences, Kwame Nkrumah University of Science and Technology. Informed consent was obtained from patients prior to sample taking. All samples were handled anonymously.

## 3. Results

### 3.1. Demographic of Patients

In total, fifty-eight clinically suspected BU patients were recruited in this study (Supplementary Materials). The median age of the participants was 17 years, with more females than males. The majority of the suspected BU patients (57%) presented ulcers, while 31%, 7% and 5% presented plaque, nodule and edema, respectively. Table 1 gives a summary of the lesion characteristics of suspected cases.

### 3.2. Quantity and Quality of DNA

**Figure 2:**
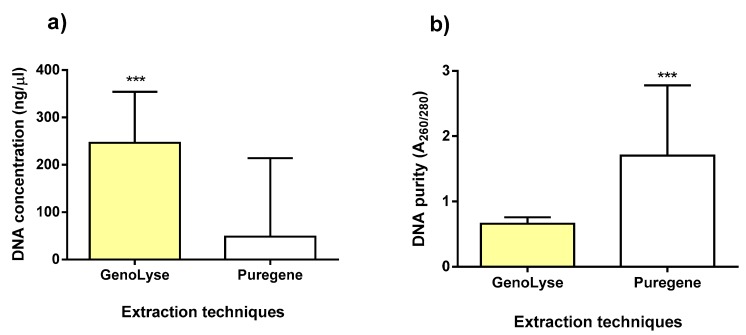
DNA concentration (**a**) and purity (**b**) of 15 clinical samples extracted with the GenoLyse and Puregene DNA extraction kit. *** represent a *p* < 0.001

The results of DNA quantity and purity for the initial 15 samples extracted with the two extraction kits—the Puregene and GenoLyse^®^ DNA extraction kit—are shown in Figure 2. A significant difference in DNA quantity (*p* < 0.001) and purity (*p* < 0.001) was obtained between the two different extraction methods, with the Puregene and Genolyse extraction technique resulting in a higher purity and DNA concentration respectively. There was no significant difference in the positivity rate between the two methods as indicated in Table 2.

### 3.3. Clinical Sensitivity and Specificity of Mu DNA GenoLyse RPA Assay

The diagnostic performance of Mu DNA GenoLyse RPA was assessed using a panel of 43 samples collected from suspected BU cases during routine care by experts. Thirty (30) samples were confirmed as BU by PCR. Twenty-six (26) out of the 30 confirmed BU samples were accurately confirmed by the Mu DNA GenoLyse and Mu RPA protocol giving a sensitivity of 87% (95% CI: 69–96). All negative 13 PCR negative samples were negative for RPA yielding a specificity of 100% (95% CI: 75–100). When qPCR was used to amplify samples extracted with Mu DNA GenoLyse protocol, the sensitivity and specificity was 73% (95% CI: 54–87) and 100% (95% CI: 75–100), respectively, compared to the Mu Puregene qPCR (Table 3).

**Figure 3:**
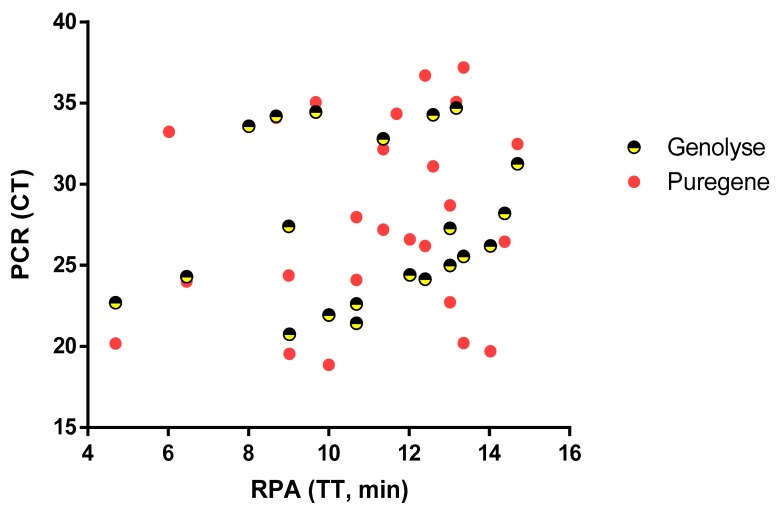
Results of 48 clinical samples extracted and amplified with both Mu DNA GenoLyse RPA protocol and Puregene qPCR protocol. No significant correlation was found between RPA (TT) and PCR (CT), even though RPA assay was very fast for some samples of high/low CT value and the vice versa. CT: cycle threshold; TT: threshold time.

Stratifying the samples by sample types (FNA and swab), the sensitivity of Mu GenoLyse RPA was 83% (95% CI: 52–98) and 89% (95% CI: 65–99) for FNA and swab samples, respectively. A 100% specificity was achieved for both sample type (Table 3). A weak positive correlation was found between threshold time (TT) of RPA and cycle threshold (CT) of qPCR results for samples extracted with Puregene (*r* = 0.23, *p* = 0.3201) and Mu DNA GenoLyse (*r* = 0.11, *p* = 0.6037) (Figure 3). There was no significant difference (*p* = 0.349) between CT of Puregene qPCR and Mu DNA GenoLyse qPCR.

## 4. Discussion

The development of a field deployable diagnostic platform for diagnosing clinically suspected BU patients is a research priority by the WHO [21]. Recently, we developed a real time recombinase polymerase amplification assay for the rapid detection of *Mycobacterium ulcerans*, the causative agent of BU [3]. However, the performance of this assay as well as other molecular assays such as LAMP in endemic communities requires simple but efficient DNA extraction techniques.

In this study, we evaluated a very fast, field adaptable and cost-effective DNA extraction technique for the extraction of Mu DNA in clinical samples using a modified GenoLyse^®^ DNA extraction procedure. We combined this DNA extraction technique with our already developed RPA assay for the effective diagnosis of suspected BU cases. The whole procedure was optimized for easy application in a mobile laboratory suitcase [19,20]. The clinical sensitivity and specificity of this procedure was 87% and 100% respectively, when compared to the routinely used Puregene DNA extraction qPCR protocol for *M. ulcerans* confirmation [16,17,18]. The diagnostic performance of this new protocol was comparable to our earlier developed Mu RPA assay on archived DNA extracted with a purification kit (the sensitivity and specificity were 88% and 100%, respectively), suggesting no negative effect of the rapid extraction on Mu RPA assay diagnostic performance. We can, therefore, safely suggest that the reduced sensitivity is as a result of the amplification efficiency of the Mu RPA. Our choice of the GenoLyse ^®^ DNA extraction Kit was supported by the fact that this kit has been successfully used to extract *Mycobacterium tuberculosis* DNA from sputum samples without any effect on downstream applications [22,23].

Different extraction procedures have been used for the extraction of Mu DNA from environmental (detritus, plant/biofilm, algae water, feces, insects and soil) and clinical samples (FNA, swab and tissue biopsy) (Table 4). 

These techniques are very efficient in extracting *M. ulcerans* DNA. However, they are labor intensive, require several pipetting steps and are limited to well-equipped laboratories not available in endemic communities. This situation limits the application of such extraction techniques at point of need. Ablordey et al. and Souza et al. evaluated the simple boiling of samples as a field-friendly DNA extraction technique that eliminates the need for labor intensive, time-consuming and costly DNA extraction procedures [7,24]. However, this technique adversely reduced the diagnostic performance of the BU LAMP assay and requires ultra-fast centrifugation, which might not be feasible in endemic communities [7,24].

The Mu DNA GenoLyse extraction protocol eliminates the need for high-speed centrifuges required for typical GenoLyse^®^ extraction procedures [22,23] or other *M. ulcerans* DNA extraction procedures (Table 4) with low-speed centrifuges which are applicable in a mobile suitcase laboratory under field conditions. Further, this protocol involved only two pipetting steps and the turnaround time from “samples in”, extraction, Mu RPA master mix preparation to amplification/detection was approximately 40 min. The centrifugation step introduced after cell lysis enables the partial purification of nucleic acid by the pelleting of cell debris and some inhibitors which might affect downstream applications. The cold chain independent nature of both GenoLyse^®^ and RPA reagents (i.e., reagents stable at room temperature for long period) provides an added advantage for the feasibility of this protocol and Mu RPA assay under field conditions. The field as used in this manuscript refers to Buruli ulcer treatment hospitals within endemic districts where patients receive care (point of need).

RPA assays have been shown to withstand many known PCR inhibitors, including hemoglobin, heparin, serum and ethanol [25]. A study showed that RPA can amplify DNA in crude prepared urine samples [26]. This advantage of RPA was demonstrated by Mu RPA assay over qPCR when both tests were run to amplify samples extracted with the Mu DNA GenoLyse protocol. Four (4) samples became negative for qPCR but positive for Mu RPA, resulting in a reduced sensitivity (73%) of Mu GenoLyse qPCR (Table 3). Samples which were positive for Puregene qPCR but negative for Mu DNA GenoLyse RPA assay could be as a result of sampling errors or the reduced amount of DNA below the detection limit of Mu RPA. If the former is true, it would suggest that the successful application of the Mu DNA GenoLyse protocol and Mu RPA assay would hinge on successful sample taking in the field. Although RPA assays can withstand PCR inhibitors, a high background DNA concentration has been demonstrated to affect the sensitivity of RPA assays [27]. This problem can be resolved by diluting DNA extracts of negative samples for Mu RPA and rerunning them. The application of this solution with regard to this study protocol should be performed with caution for very typical BU cases to prevent the wastage of reagents and the contamination of workspace, which could result in false positive results. An introduction of a purification step in the Mu DNA GenoLyse extraction protocol will also help increase the sensitivity of the Mu DNA GenoLyse qPCR protocol. The low sensitivity of the combined protocol also suggests that negative results of clinically suspected cases need to be confirmed with qPCR. Notwithstanding, the application of the Mu DNA GenoLyse protocol and the Mu RPA assay in a mobile suitcase laboratory still needs to be evaluated in local district hospitals or clinics in order to determine the field applicability or adaptability of these protocols.

## 5. Conclusions

We have evaluated a simple, rapid and sensitive protocol for the extraction of *M. ulcerans* DNA from clinical samples of suspected BU patients. The unsophisticated extraction procedure coupled with the minimal technical demand of the Mu RPA assay facilitates application in a mobile suitcase laboratory [19,20]. This setup will enable the early detection of Buruli ulcer-suspected lesions directly at the point of need, allowing the prompt treatment of confirmed cases.

## Figures and Tables

**Table 1:** Demographic and clinical characteristics of Buruli ulcer (BU)-suspected cases used in the study.

Parameters	No. (%) of Total Lesions (*n* = 58)
**Sex**	
Male	23 (40)
Female	35 (60)
**Sample Type**	
Swab	33 (57)
FNA	25 (43)
**Age in Years**	
Median (IQR)	17 (8-39)
**Type of Lesion**	
Ulcer	33 (57)
Nodule	4 (7)
Plaque	18 (31)
Edema	3 (5)
**Category of Lesion**	
I	27 (47)
II	16 (28)
III	15 (26)

**Table 2:** Number of positive clinical samples analyzed with the GenoLyse and Puregene extraction methods.

	No. Positive/No. Clinical Confirmed as BU (% Positivity)	
	GenoLyse	Puregene *
Polymerase chain reaction (PCR)	12/15 (80)	12/15 (80)
Recombinase polymerase amplification (RPA)	12/15 (80)	12/15 (80)

* The Puregene extraction method was used as the standard extraction method.

**Table 3:** Diagnostics performance of the Mu DNA GenoLyse RPA protocol compared to qPCR.

			Puregene DNA qPCR *		Total	Sensitivity %	Specificity %	PPV %	NPV %
			+ve	−ve		(95% CI)	(95% CI)	(95% CI)	(95% CI)
**Mu DNA GenoLyse qPCR**		+ve	22	0	22	73 (54–87)	100 (75–100)	100 (85–100)	62 (38–81)
		−ve	8	13	21				
**Mu DNA GenoLyse RPA**	Swab	+ve	16	0	16	89 (65–99)	100 (66–100)	100 (79–100)	82 (48–98)
		−ve	2	9	11				
	FNA	+ve	10	0	10	83 (52–98)	100 (40–100)	100 (69–100)	67 (22–96)
		−ve	2	4	6				
	Total	+ve	26	0	26	87 (69–96)	100 (75–100)	100 (87–100)	75 (50–93)
		−ve	4	13	17				

+ve: positive, -ve: negative, PPV: positive predictive value, NPV: negative predictive value, and FNA: fine-needle aspirate. * Puregene DNA extract was used as the DNA.

**Table 4:** Comparison of different extraction protocols for Buruli ulcer clinical samples and environmental samples.

Reference	Kit/Extraction Method	Kit-Producing Company	Purification Method	Time Needed (min) ^a^	Samples	Overnight Incubation Step	Heating Step (37–70 °C)	Proteinase K	Centrifugation	Pipetting Steps (≥10)	Costs per Reaction (€)
[9,10]	One-tube cell lysis		silica-cellulose membrane columns	190	tissue and environmental specimens	+	+	−	+	+	Unknown
[10]	FastPrep^®^ SPINKit	MP Biomedicals, Brussels, Belgium	silica filter column	60	tissue and environmental specimens	−	+	−	+	+	4.42
[10]	Modified Boom procedure		diatomaceous earth	182	tissue and environmental specimens	+	+	+	+	+	Unknown
[10]	Maxwell^®^ 16 kit	Promega, Leiden, Netherlands	MagneSil paramagnetic particles	70	tissue and environmental specimens	+	+	+	+	5 ^#^	4.61
[13,14]	Guanidinium thiocyanate (GuSCN)-diatoms method		diatom	45	FNA, swabs and tissue biopsies	+	+	+	+	+	Unknown
[16]	Puregene Extraction Kit	Qiagen, Hilden, Germany	chemical (isopropanol +glycogen)	300	FNA, swabs and tissue biopsies	+	+	+	+	+	2
[7,24]	Boiling method		centrifugation	15	FNA and swabs	−	−	−	+	2	Unknown
This study	Mu DNA GenoLyse	Hain Lifescience GmbH, Germany	centrifugation	15	FNA and swabs	−	−	−	+	2	0.8

^a^ Time excluding the overnight incubation; ^#^ automated DNA extraction procedure; + is employed and − is not employed in the respective protocol.

## Supplementary Materials

The following are available online at https://www.mdpi.com/2075-4418/9/4/204/s1.
